# The Homogalacturonan Deconstruction System of Paenibacillus amylolyticus 27C64 Requires No Extracellular Pectin Methylesterase and Has Significant Industrial Potential

**DOI:** 10.1128/AEM.02275-19

**Published:** 2020-06-02

**Authors:** Christian Keggi, Joy Doran-Peterson

**Affiliations:** a Department of Microbiology, University of Georgia, Athens, Georgia, USA; University of Toronto

**Keywords:** *Paenibacillus*, pectic enzymes, pectinases

## Abstract

Paenibacillus amylolyticus 27C64, a Gram-positive bacterium with diverse plant cell wall polysaccharide deconstruction capabilities, was isolated previously from an insect hindgut. Previous work suggested that this organism’s pectin deconstruction system differs from known systems in that its sole pectin methylesterase is cytoplasmic, not extracellular. In this work, we have characterized the specific roles of key extracellular pectinases involved in homogalacturonan deconstruction, including four pectate lyases and one pectin lyase. We show that one newly characterized pectate lyase, PelC, has a novel substrate specificity, with a lower *K_m_* for highly methylated pectins than for polygalacturonic acid. PelC works synergistically with PelB, a high-turnover exo-pectate lyase that releases Δ4,5-unsaturated trigalacturonate as its major product. It is likely that PelC frees internal stretches of demethylated homogalacturonan which PelB can degrade. We also show that the sole pectin lyase has a high *k*_cat_ value and rapidly depolymerizes methylated substrates. Three cytoplasmic GH105 hydrolases were screened for the ability to remove terminal unsaturated galacturonic acid residues from oligogalacturonide products produced by the action of extracellular lyases, and we found that two are active on demethylated oligogalacturonides. This work confirms that efficient homogalacturonan deconstruction in P. amylolyticus 27C65 does not require extracellular pectin methylesterase activity. Three of the extracellular lyases studied in this work are also thermostable, function well over a broad pH range, and have significant industrial potential.

**IMPORTANCE** Pectin is an important structural polysaccharide found in most plant cell walls. In the environment, pectin degradation is part of the decomposition process that turns over dead plant material and is important to organisms that feed on plants. Industrially, pectinases are used to improve the quality of fruit juices and can also be used to process coffee cherries or tea leaves. These enzymes may also prove useful in reducing the environmental impact of paper and cotton manufacturing. This work is significant because it focuses on a Gram-positive bacterium that is evolutionarily distinct from other well-studied pectin-degrading organisms and differs from known systems in key ways. Most importantly, a simplified extracellular deconstruction process in this organism is able to break down pectins without first removing the methyl groups that inhibit other systems. Moreover, some of the enzymes described here have the potential to improve industrial processes that rely on pectin deconstruction.

## Abstract (precis)

Pectin is an important structural polysaccharide found in most plant cell walls. In the environment, pectin degradation is part of the decomposition process that turns over dead plant material and is important to organisms that feed on plants. Industrially, pectinases are used to improve the quality of fruit juices and can also be used to process coffee cherries or tea leaves. These enzymes may also prove useful in reducing the environmental impact of paper and cotton manufacturing. This work is significant because it focuses on a Gram-positive bacterium that is evolutionarily distinct from other well-studied pectin-degrading organisms and differs from known systems in key ways. Most importantly, a simplified extracellular deconstruction process in this organism is able to break down pectins without first removing the methyl groups that inhibit other systems. Moreover, some of the enzymes described here have the potential to improve industrial processes that rely on pectin deconstruction.

## INTRODUCTION

Pectins are a complex family of galacturonic acid (GalA)-rich polysaccharides present in virtually all plant cell walls, structures that store the majority of the 55 billion tons of carbon fixed by plants in terrestrial ecosystems each year (1). Pectic polysaccharides are abundant in the primary cell walls and middle lamellae of most plants, where they play a number of vital roles (2, 3). The pectin network is interwoven with and covalently linked to other matrix polysaccharides (4–6), cellulose (7), and lignin (8) and is structurally important. In *Arabidopsis*, disruption of normal pectin biosynthesis results in dwarfism and brittle stems (9). Pectin is the main component of the outermost cell wall layer, the middle lamella, where it is responsible for cell-cell adhesion (2, 3, 10). Pectin has also been implicated in a number of other important plant functions, including cell defense and development (10). This family of polysaccharides includes several structurally distinct types, of which homogalacturonan (HG) and rhamnogalacturonan I (RG-I) are the two most abundant (10). HG accounts for approximately 65% of pectin and comprises a linear 1,4-α-d-GalA backbone that can be methylated at the C-6 carboxyl or acetylated at the O-2 or O-3 position. While methylation is often extensive, in some cases occurring on >90% of the GalA residues, acetylation is typically minimal, except in the pectins of a few specific plant species (10). RG-I has an alternating GalA-rhamnose backbone that may also be acetylated and has variable arabinose- and galactose-rich side chains attached to 20 to 80% of the rhamnosyl residues (10).

The mechanisms of microbial pectin deconstruction have been of interest over the past few decades, since pectinases are important virulence factors for some Gram-negative plant pathogens and are also useful for fruit juice processing. Specifically, three genera of plant pathogens within the *Enterobacteriaceae*—*Pectobacterium*, *Dickeya*, and *Erwinia*—have well-studied pectin deconstruction systems (11), while enzymes from various *Aspergillus* species that are active under acidic conditions are commonly used to improve fruit juice yields, reduce the viscosity of the extracted juice, and clarify the final product (12, 13). More recently, pectin has been identified as an important contributor to biomass recalcitrance in feedstocks used for lignocellulosic ethanol production, such as pectin-rich agricultural wastes (14), herbaceous plant tissues (15–17), and woody plant material (18–20). Additionally, deletion of a pectinase gene cluster from an organism that can normally grow on unmodified plant material, Caldicellulosiruptor bescii, results in a growth defect when *Arabidopsis*, switchgrass, or poplar wood is supplied as the growth substrate (21). This suggests that efficient pectin deconstruction is necessary to eliminate or reduce the severity of thermochemical pretreatment steps. Better understanding of microbial pectin deconstruction, especially in organisms phylogenetically distinct from *Enterobacteriaceae* and *Aspergillus*, may improve the efficiency and reduce the cost of plant cell wall deconstruction for biofuel or chemical production and may also help identify novel enzymes with other industrial uses. Bacterial enzymes often have alkaline optimum pH ranges and are of particular interest for retting and degumming plant fibers in textile manufacturing, removing pulp from coffee cherries, and processing tea leaves (13, 22). These bacterial enzymes also facilitate the removal of noncellulosic polysaccharides from paper pulp or cotton, thereby reducing the need for strongly basic processing steps and eliminating problems associated with alkaline wastewater (13, 22).

Paenibacillus amylolyticus 27C64 is a Gram-positive bacterium that falls into a taxonomic group distinct from those of the other well-studied pectinolytic bacteria. It was originally isolated from the microbial hindgut community of an aquatic crane fly (Tipula abdominalis) larva, where it is partly responsible for the breakdown of plant material ingested by the insect (23). This isolate displayed a wide range of plant cell wall polysaccharide deconstruction capabilities when screened on differential media (23), and two of its pectate lyases have been characterized (24). These two enzymes, PelA and PelB, retained an unusual amount of activity on highly methylated pectins, which are normally poor substrates for pectate lyases (24). Subsequent analysis of the carbohydrate-active enzymes (CAZymes) present in the genome revealed a remarkable diversity of CAZymes, including 10 enzymes believed to be capable of deconstructing HG (25). The regulation of these putative pectinases when P. amylolyticus was grown with different pectic substrates provided some insight into the functional role of each enzyme. Six enzymes with predicted signal peptides, including a pectin acetylesterase that removes O-2 or O-3 acetylation (Pae), four pectate lyases that cut at unmethylated GalA residues (PelA, PelB, PelC, and PelD), and one pectin lyase that cuts at methylated sites (Pnl), seemed to be responsible for extracellular HG deconstruction (Table 1). Two putatively cytoplasmic GH105 hydrolases (YteR and YteZ), which likely remove terminal unsaturated GalA residues from HG-derived oligosaccharides, were identified, along with a pectin methylesterase (Pem; removes C-6 methylation) and a polygalacturonase (Peh; cleaves unmethylated substrates by hydrolysis) (Table 1). Protein names, in lieu of locus tags generated by annotation software that were referenced previously, are assigned here for clarity and are based on sequence identity to biochemically similar enzymes where possible. Despite some differences in the regulation of these enzymes (25), significant ambiguity in the precise biochemical role of each enzyme exists. For example, the utility of four catalytically redundant pectate lyases was unclear.

**TABLE 1:** Genes characterized and discussed in this study

Protein category and gene	Protein	CAZy assignment	Product	Size (kDa)
Extracellular proteins				
pamy_2972	PelA	PL3	Pectate lyase	20.7
pamy_4343	PelB	PL1	Pectate lyase	48.0
pamy_1763	PelC	PL9	Pectate lyase	43.3
pamy_4669	PelD	PL10	Pectate lyase	31.6
pamy_2278	Pnl	PL1	Pectin lyase	35.2
pamy_4060	Pae	CE12	Pectin acetylesterase	38.1
Cytoplasmic proteins				
pamy_1066	YteR	GH105	Unsaturated galacturonidase	42.2
pamy_4272	YteZ	GH105	Unsaturated galacturonidase	42.1
pamy_5356	YesR	GH105	Unsaturated RG hydrolase	39.2
pamy_4273	Pem	CE8	Pectin methylesterase	51.0
pamy_82	Peh	GH28	Polygalacturonase	58.7

It has been noted previously that this system apparently lacks an extracellular pectin methylesterase, a finding that stands in stark contrast to those for Gram-negative (11) and fungal (26) systems and perhaps explains the high activities of PelA and PelB on methylated substrates (24). In this study, we confirm that the extracellular pectinases of P. amylolyticus 27C64 are able to break down methylated HG without the aid of an extracellular pectin methylesterase, and we provide a model of HG deconstruction that may be applicable to other Gram-positive bacteria. We also identify elements of the system that have significant industrial potential.

(This research was conducted by Christian Keggi in partial fulfillment of the requirements for a Ph.D. from the University of Georgia, 2019 [27].)

## RESULTS

### Protein purification.

All five extracellular lyases (PelA, PelB, PelC, PelD, Pnl) had their polyhistidine-rubredoxin affinity tags removed and were purified until no other bands were clearly visible on a Coomassie blue-stained SDS-PAGE gel (see Fig. S1 in the supplemental material). Polyhistidine-rubredoxin tags were not removed from the cytoplasmic hydrolases (YteR, YteZ, YesR), and very few contaminants were present on SDS-PAGE gels (Fig. S1). The relative mobilities of the proteins on SDS-PAGE gels were consistent with their theoretical molecular weights.

### Characterization of pectic substrates.

The commercial substrates used had very different degrees of methylation (DM), ranging from 0 to 81.7% (Table S1). Most DM values were consistent with the product labeling if a degree of esterification was specified, except in one case (CP_90). Acetylation was minimal across all the substrates, with the highest degree of acetylation (DA), for AP_Sigma, reaching 3.4%. Most substrates were high-molecular-weight pectins, although CP_ICN and RG_P-A had low-molecular-weight peaks as well. CP_90 was notably different from the other citrus pectins: it included a broad mix of high- and low-molecular-weight fragments.

### Determination of optimum reaction conditions.

The three lyases newly characterized in this study were PelC, PelD, and Pnl. All three worked optimally in a neutral-to-alkaline pH range (Fig. S2). PelC had optimal activity at pH 10.0 and 55°C, with 0.1 to 1.0 mM CaCl_2_ included in the reaction buffer. Optimal activity for PelD was observed at pH 9.0 and 45°C in the presence of 1 mM CaCl_2_. Optimal activity for Pnl was observed between pH 9 and 10 at a temperature of 55°C. Although 1 mM CaCl_2_ slightly increased the activity of Pnl, it was not essential, and addition of 1 mM EDTA did not affect activity. In contrast, no PelC or PelD activity could be detected when 1 mM EDTA was included in the reaction buffer (data not shown). The optimum reaction conditions for PelA and PelB have been described elsewhere (24), but the temperature optimum for PelB activity was reevaluated, since new equipment enabled measurements at higher temperatures. The new optimum temperature for PelB activity is 70°C.

### Substrate specificity.

**FIG 1:**
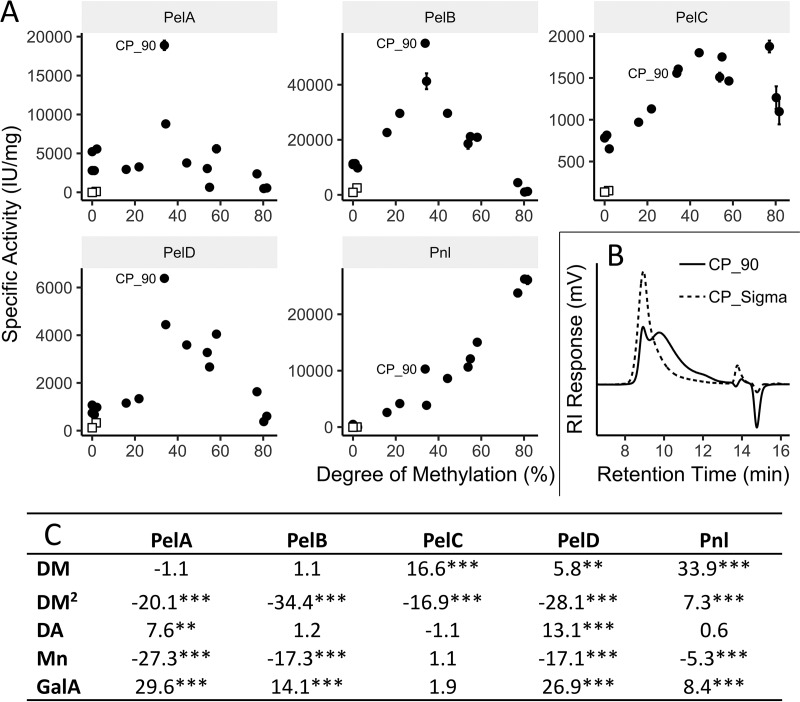
Correlation between key characteristics of commercial pectins and the activity of each extracellular lyase. (A) Scatter plots show the specific activity of each enzyme as a function of substrate methylation, the most important substrate characteristic (*n* = 3). Open squares represent rhamnogalacturonan I substrates. (B) Size exclusion elution profiles are provided for a typical high-molecular-weight pectin, CP_Sigma, and the lowest-molecular-weight pectin, CP_90. (C) Standardized parameter estimates from linear models comparing the relative activity of each enzyme to the degree of methylation (DM), second-order degree of methylation (DM^2^), degree of acetylation (DA), number-average molecular weight (*M*_n_), and galacturonic acid content (GalA) of each pectin. Asterisks denote significant differences (*, *P < *0.05; **, *P < *0.01; ***, *P < *0.001).

The activity of each extracellular lyase was evaluated on 17 different commercially available pectins, and multiple linear models were used to determine the impact of methylation, acetylation, molecular weight, and GalA content on activity (Table S2). All of the enzymes were specific to homogalacturonan and had no measurable activity on rhamnogalacturonan. Overall, substrate methylation had the greatest impact on activity. Initial reaction rates for PelA, PelB, and PelD were highest on moderately methylated pectins, while PelC and Pnl displayed more activity on highly methylated substrates than on minimally or moderately methylated substrates (Fig. 1A). Except for PelC, the activity of each enzyme was significantly higher on lower-molecular-weight substrates (*P < *0.001), but the magnitude of this effect was greatest for PelA (Fig. 1C). In fact, the molecular weight of the substrate had a greater impact on PelA activity than substrate methylation. Acetylation did have an impact on the activities of PelA and PelD (*P < *0.01), but the magnitude of the effect was small, and it was not a major predictor of activity. The GalA content of each substrate was included as a model term to control for its effect, and with the exception of PelC, all of the enzymes displayed significantly higher activities with higher GalA contents (*P < *0.001).

### Kinetics.

Three high-molecular-weight pectins with various degrees of methylation were selected to determine the kinetic values of each enzyme: PGA-C (0% methylated), CP_Sigma (55% methylated), and CP_85-C (80% methylated). Generally, the four pectate lyases had similar *k*_cat_ values on each substrate, but *K_m_* values differed (Table 2). PelA had low *K_m_* values on PGA-C and CP_Sigma, but its *K_m_* on CP_85-C was more than an order of magnitude higher. PelB, PelC, and PelD had the lowest *K_m_* values on CP_Sigma. However, while PelB and PelD had *K_m_* values for CP_85-C that were an order of magnitude higher than those for PGA-C, PelC had a lower *K_m_* for CP_85-C than it had for PGA-C. The pectin lyase, Pnl, had much higher affinity for methylated substrates than for PGA-C, and its turnover rate was hindered by low methylation. PelA and PelD were inhibited by high concentrations of the substrates polygalacturonic acid (PGA) and CP_Sigma. Some deviation from the expected Michaelis-Menten curve was observed for PGA with PelB, PelC, and PelD (Fig. S3). It is possible that this deviation is an artifact of substrate binding to assay materials, since PGA is anionic and is supplied in its acidic (not salt) form, unlike the other two substrates. The viscosity of the assay solutions also increases with increasing substrate concentration, and demethylated substrates have a greater tendency to thicken in the presence of calcium, which is required for pectate lyase activity. This effect may also explain the substrate inhibition effects seen with some enzymes and the deviations from the normal Michaelis-Menten curve. Alternatively, the processivity of these enzymes on completely demethylated substrates may cause a deviation from the Michaelis-Menten model. Despite these limitations, the *K_m_* values still effectively summarize the relative affinities of each enzyme for the three different substrates and are provided for comparison to other enzymes for which Michaelis-Menten kinetic values have been published.

**TABLE 2:** Kinetic parameters of each extracellular lyase on three pectic substrates

Enzyme	Parameter (unit of measurement)	Value for:		
		PGA-C	CP_Sigma	CP_85-C
PelA	*K_m_* (mg/ml)	0.24 ± 0.06	0.43 ± 0.10	8.44 ± 2.58
	*V*_max_ (IU/mg)	39.7 ± 6.38	14.7 ± 2.42	32.1 ± 8.28
	*k*_cat_ (s^−1^)	(4.94 ± 0.79) × 10^4^	(1.83 ± 0.30) × 10^4^	(3.99 ± 1.03) × 10^4^
	*k*_cat_/*K_m_* (ml mg^−1^ s^−1^)	2.04 × 10^5^	4.22 × 10^4^	4.73 × 10^3^
PelB	*K_m_* (mg/ml)	2.48 ± 0.95	0.55 ± 0.09	50.1 ± 140
	*V*_max_ (IU/mg)	458 ± 110	302 ± 19.6	745 ± 2,024
	*k*_cat_ (s^−1^)	(1.32 ± 0.32) × 10^6^	(8.69 ± 0.56) × 10^5^	(2.14 ± 5.83) × 10^6^
	*k*_cat_/*K_m_* (ml mg^−1^ s^−1^)	5.30 × 10^5^	1.57 × 10^6^	4.28 × 10^4^
PelC	*K_m_* (mg/ml)	1.02 ± 0.23	0.04 ± 0.00	0.41 ± 0.06
	*V*_max_ (IU/mg)	15.1 ± 1.55	9.68 ± 0.471	12.8 ± 0.610
	*k*_cat_ (s^−1^)	(3.94 ± 0.41) × 10^4^	(2.52 ± 0.12) × 10^4^	(3.32 ± 0.16) × 10^4^
	*k*_cat_/*K_m_* (ml mg^−1^ s^−1^)	3.84 × 10^4^	5.49 × 10^5^	8.02 × 10^4^
PelD	*K_m_* (mg/ml)	3.05 ± 1.95	0.89 ± 0.55	33.6 ± 159
	*V*_max_ (IU/mg)	304 ± 131	185 ± 81.1	531 ± 2,400
	*k*_cat_ (s^−1^)	(5.77 ± 2.48) × 10^5^	(3.51 ± 1.54) × 10^5^	(1.01 ± 4.55) × 10^6^
	*k*_cat_/*K_m_* (ml mg^−1^ s^−1^)	1.89 × 10^5^	3.94 × 10^5^	2.99 × 10^4^
Pnl	*K_m_* (mg/ml)	7.61 ± 9.26	0.07 ± 0.00	0.13 ± 0.00
	*V*_max_ (IU/mg)	11.91 ± 11.91	59.27 ± 0.676	144.6 ± 1.894
	*k*_cat_ (s^−1^)	(2.52 ± 2.52) × 10^4^	(1.25 ± 0.01) × 10^5^	(3.05 ± 0.04) × 10^5^
	*k*_cat_/*K_m_* (ml mg^−1^ s^−1^)	3.30 × 10^3^	1.57 × 10^6^	2.21 × 10^6^

### Analysis of reaction products and mode of action.

**FIG 2:**
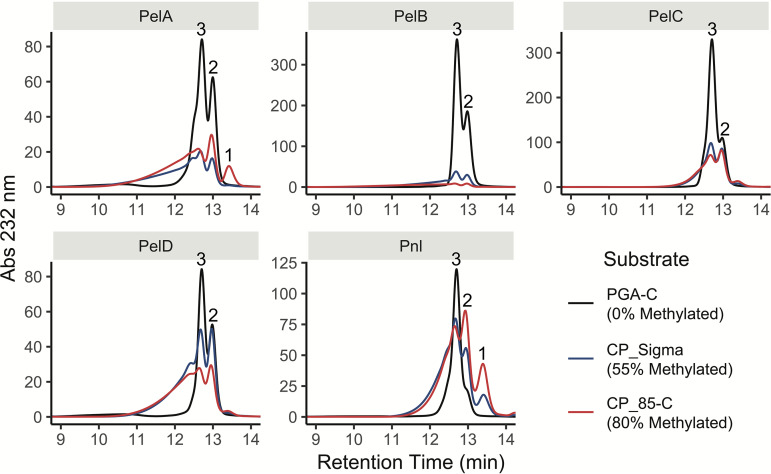
Analysis of products released by the action of each extracellular lyase on three different pectins. Reactions were allowed to run to completion; then digestion products were separated by high-performance size exclusion chromatography and were detected by absorbance at 232 nm. Numbers above major peaks indicate the associated degree of polymerization and were determined using mono-, di-, and tetragalacturonic acid size standards.

The products formed by the action of each extracellular lyase on the same three pectins used for kinetic assays were analyzed after the reactions had been allowed to run to completion (Fig. 2). All four pectate lyases released Δ4,5-unsaturated trigalacturonate as their major product on PGA-C but also generated Δ4,5-unsaturated digalacturonate. When these enzymes acted on methylated substrates, a mix of higher-molecular-weight oligogalacturonides and lower overall product release was observed. However, PelB was hindered by methylation to a much greater extent than the other pectate lyases. The pectin lyase released Δ4,5-unsaturated digalacturonate as the major product on CP_85-C but an unsaturated trisaccharide as the major product on CP_Sigma and PGA.

**FIG 3:**
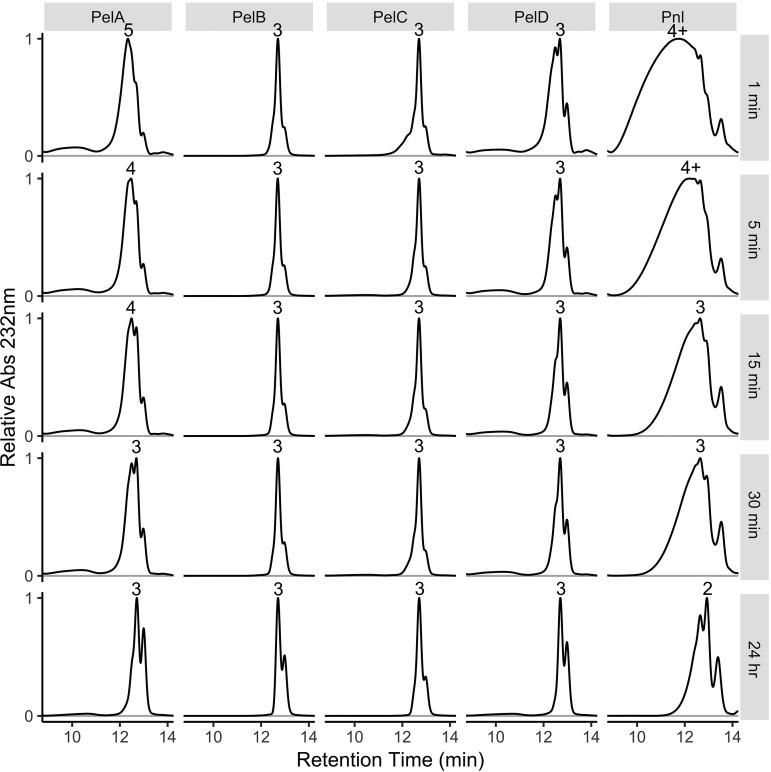
Mode of action of extracellular pectate lyases. Intermediate reaction products produced by the actions of PelA, PelB, PelC, and PelD on polygalacturonic acid (0% methylated) or by the action of Pnl on 80% methylated citrus pectin were analyzed by high-performance size exclusion chromatography. Absorbance at 232 nm was used to detect products. All chromatograms were scaled to fill the plot area so that product distributions could be clearly visualized. Time points are given on the right. Numbers above peaks indicate the degree of polymerization associated with the major product at a given time point and were determined by comparison to mono-, di-, and tetragalacturonic acid size standards.

Intermediate reaction products of PGA digestion reactions with each pectate lyase or of CP_85-C digestion with the pectin lyase were evaluated at five time points. Pnl initially released exclusively very large products, which were later degraded primarily to unsaturated digalacturonate (Fig. 3). In contrast, PelA and PelD initially produced a mixture of short oligogalacturonides and some larger products that were later degraded to unsaturated tri- and disaccharides. PelC released mostly unsaturated trigalacturonate, but larger products were observed at the first time point. PelB consistently released a mixture of unsaturated tri- and digalacturonate.

### Synergy between extracellular lyases.

**FIG 4:**
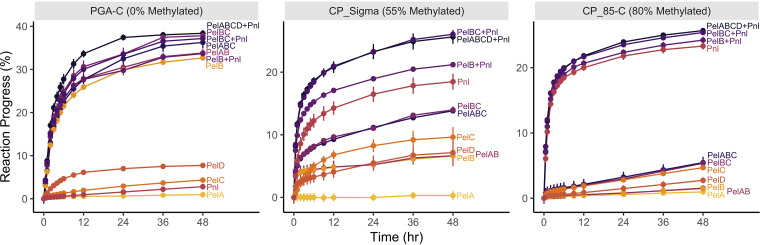
Effects of extracellular lyase combinations on the digestion of three different pectins. Each enzyme was included at a 1 nM concentration, and the reactions were monitored by tracking the absorbance at 232 nm. The *y* axis represents reaction progress as a percentage of the theoretical maximum absorbance, calculated using the galacturonic acid content of each substrate and the molar extinction coefficients of the products. Thirty-three percent progress is the maximum achievable assuming that all available galacturonic acid is broken down into a trimer.

The abilities of enzyme combinations to completely deconstruct PGA-C, CP_Sigma, and CP_85-C were evaluated using very low enzyme concentrations (1 nM). Under these conditions, PelA had very little activity compared to those of the other enzymes. PelB accounted for most of the PGA-C deconstruction, while Pnl was the most effective enzyme on the highly methylated substrate CP_85-C (Fig. 4). In contrast, CP_Sigma (55% methylated) required both pectate and pectin lyases for complete deconstruction. On this moderately methylated substrate, PelB and PelC worked synergistically, achieving a higher percentage of deconstruction together than either did separately.

### Roles of cytoplasmic GH105 hydrolases.

**FIG 5:**
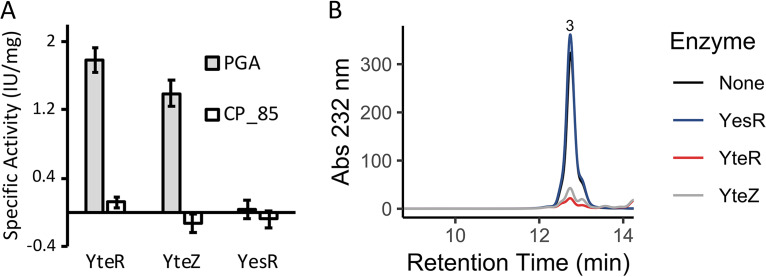
Activities of the three GH105 hydrolases on unsaturated oligogalacturonides. PGA (0% methylation) was digested with PelB and PelC, while CP_85-C (80% methylation) was digested with Pnl, to generate mixed oligogalacturonides. Digested substrates were heat inactivated and cooled before the addition of each GH105 enzyme. (A) Bars represent the specific activity of each enzyme, measured as the decrease in absorbance at 232 nm (*n* = 3). (B) High-performance size exclusion elution profiles of reactions allowed to run to completion were monitored at 232 nm as well. The number above the major peak indicates its degree of polymerization as determined by comparison to oligogalacturonide size standards.

The three GH105 hydrolases that were previously shown to be upregulated on different pectic substrates (25) were screened for the ability to remove terminal Δ4,5-unsaturated GalA residues from the product mixtures generated by the digestion of PGA-C with PelB and PelC or by Pnl digestion of CP_85-C. Both YteR and YteZ, enzymes that were upregulated on substrates containing demethylated HG and methylated HG, respectively, were active on products released from PGA but had no activity on methylated fragments (Fig. 5A). YesR, the GH105 enzyme that was upregulated on RG-I only, had no activity on any of the HG digestion products. High-performance size exclusion chromatography (HPSEC) analysis of these GH105 digestion reactions confirmed that breakdown of the unsaturated trisaccharide released by PelC and PelB was the source of the decrease in absorbance used to assay the enzymes (Fig. 5B).

## DISCUSSION

### Role of each extracellular depolymerase.

Prior to this work, the differential regulation of each enzyme in this system had been investigated, and it was known that PelA, PelB, and PelC were upregulated whenever pectins were used as a carbon source, that PelD was upregulated only when RG-I was supplied for growth, and that Pnl was constitutively expressed (25). An extracellular pectin acetylesterase that had a high degree of sequence similarity to characterized enzymes had also been identified (25). Despite some differences in regulation, the specific roles of each of the five extracellular depolymerases remained unclear, especially those for the three pectate lyases that had similar expression patterns (PelA, PelB, and PelC). Correlation between pectin characteristics and the activity of each enzyme revealed some key differences. As is expected for a pectin lyase, Pnl had the most activity on highly methylated substrates, a reflection of the fact that these enzymes cut efficiently only at methylated sites. It was noted previously that PelA and PelB have unusually high activities on methylated substrates (24), an unexpected characteristic of this class of enzymes, which typically cut only at unmethylated sites. With the larger set of substrates used in this study, we confirmed the previous finding and added that PelB has significantly higher activity on moderately methylated substrates than on completely demethylated substrates (Fig. 1A and C). This is true as well for PelD, an enzyme newly described in this study. PelC, the other newly described enzyme, is even more unusual in that it has a higher initial reaction rate on the highly methylated pectins than on demethylated substrates. Taken together, these results reveal that this system is well adapted to working on methylated substrates, even though four of the five enzymes cannot cut at methylated sites. All of the enzymes, except PelC, also show a significant preference for lower-molecular-weight substrates, but this effect is most pronounced for PelA, where substrate molecular weight predicts activity better than substrate methylation. The strength of this preference led to PelA having much higher activity on CP_90, the lowest-*M*_n_ substrate, than on any other pectin (Fig. 1A and B).

Kinetic experiments told a similar but more complete story, with PelA, PelB, and PelD all having much lower *K_m_* values on PGA-C and CP_Sigma than on the highly methylated substrate CP_85-C (Table 2). In other words, these three pectate lyases were not hindered by moderate methylation, functioning effectively on all but the most highly methylated substrates. Comparison of the unmethylated substrate PGA and the moderately methylated substrate CP_Sigma showed that PelB and PelD had slightly higher affinity for 55% methylation than for 0%, but PelA behaved in the opposite manner. The *K_m_* values for PelC, however, were very unusual. This enzyme not only retained activity on highly methylated substrates; it was actually hindered by a lack of methylation. The *K_m_* on 55% methylated pectin was 10-fold lower than that on the 85% methylated substrate and was highest on the demethylated substrate PGA. This finding implies that both methylated and unmethylated sites are important for PelC binding and suggests that this enzyme specifically targets the junction of methylated and demethylated regions.

Analysis of reaction products showed that Δ4,5-unsaturated trigalacturonate is the major product released by all four pectate lyases and that a lesser amount of unsaturated digalacturonate is also present (Fig. 2). Pnl produced a similar mixture with relatively more digalacturonate. Tracking of these samples over time revealed that Pnl is a true endo-type pectin lyase, since it initially produced very large fragments (Fig. 3). PelA, PelC, and PelD also cut at internal sites but produced a mixture of short oligosaccharides at earlier time points instead of primarily very large fragments. However, PelB produced a homogeneous product consistent with that of an exo-type enzyme (a processive enzyme that binds at the end of a polysaccharide). This conclusion is supported by the very small amount of product released from 80% methylated citrus pectin; if PelB could not access internal unmethylated stretches, the degree of reaction completion would be severely reduced.

Collectively, these data identify specific roles for each enzyme. PelB, as an exo-type enzyme with a very high *k*_cat_, is effective at rapidly saccharifying unmethylated stretches of HG. PelC, with a *k*_cat_ 2 orders of magnitude lower than that of PelB, is not as effective at breaking down demethylated stretches of GalA residues but can bind at the interface of methylated and unmethylated regions to free internal stretches of unmethylated residues for PelB. PelA has the lowest *K_m_* overall on PGA-C and may be useful when substrate concentrations are very low. It is also very effective on smaller substrates. Pnl, as the only enzyme able to cut at demethylated sites, is responsible for the deconstruction of methylated regions and works rapidly with a high *k*_cat_ on 80% methylated pectin. PelD, which is upregulated only on RG-I, does not have a clear role in HG deconstruction based on these experiments.

### Synergy between extracellular enzymes.

It is clear that PelB is largely responsible for the deconstruction of demethylated homogalacturonan, since it is able to break down unmethylated polygalacturonic acid almost as well as all five enzymes combined (Fig. 4). Similarly, Pnl seems chiefly responsible for the depolymerization of highly methylated substrates. However, on the moderately methylated substrate CP_Sigma, the combination of PelB and Pnl is not as effective as all five enzymes together. PelC, which can make internal demethylated regions available to the exo-type enzyme PelB, is required for complete deconstruction. Under the conditions used (low enzyme concentration, 0.25 mg/ml substrate) both PelA and PelD could be dropped from the all-enzyme mixture without compromising the rate of deconstruction or the final degree of completion. For PelD, this is likely because it is involved in removing HG regions from rhamnogalacturonan I or II. In contrast, PelA is most likely involved in HG deconstruction based on its regulation, but it may be important for the solubilization of fragments from cell walls or when substrate concentrations are very low, both possibilities that were not explored in this work.

### Cytoplasmic breakdown of pectic oligosaccharides.

The previous findings that YteR was upregulated when any pectin was supplied for growth, but that YteZ was upregulated only when methylated substrates were supplied, led to the hypothesis that YteZ may be specific to methylated substrates (25). YesR was upregulated only on RG-I, and it was assumed to be an unsaturated rhamnogalacturonyl hydrolase. Here we see that both YteR and YteZ were active on unsaturated HG-derived oligosaccharides, but, as expected, YesR was not (Fig. 5A). Interestingly, both active enzymes broke down only the demethylated fragments, despite the fact that YteZ was upregulated on methylated substrates. Such redundancy is necessary because this catabolic function is required even if only very highly methylated HG, which would not induce YteR activity, is present. Because both of these hydrolases work on the same substrate, methylated fragments would have to be demethylated by the putative cytoplasmic pectin methylesterase (Pem) first (25). A putative cytoplasmic polygalacturonase (Peh) that is also encoded in the genome likely breaks down the saturated demethylated digalacturonate remaining after the action of YteR and YteZ.

### Significance of findings.

The clearest difference between this homogalacturonan deconstruction system and those described for other organisms is that efficient and complete HG breakdown does not require an extracellular pectin methylesterase. Instead, the only pectin methylesterase here appears to be cytoplasmic. This has created selective pressure for a pectin lyase with a high *k*_cat_ and for the unique substrate specificity of PelC. Pairing this with the very high turnover rate of PelB, we conclude that these three enzymes represent a simplified set of pectinolytic enzymes that can completely degrade pectins and have significant industrial potential. In fact, since most natural pectins are moderately or highly methylated, Pnl alone is likely sufficient for many applications where only initial size reduction is required, such as removing pectic haze in beverages, reducing the viscosity of pectin-rich solutions, eliminating the foaming potential of powdered tea beverages, or removing pectinaceous pulp from coffee cherries and ramie fibers (12, 13). When evaluating potential synergy between these enzymes, we noted that reactions with PelB alone, Pnl alone, or PelBC plus Pnl were nearly half complete within 3 h despite the low concentration of substrate (initially 0.25 mg/ml) and the very low enzyme concentration (1 nM). This 1 nM concentration equates to just 1 g of each purified enzyme per 21,000 to 28,000 liters. Additionally, these experiments were carried out under simple buffer conditions at room temperature, but both PelC and Pnl are fairly thermostable, with an optimum temperature of 55°C, while PelB is very thermostable, with maximal activity at 70°C. Pnl also retains activity at cooler temperatures, working at 13% of its maximal activity at 10°C. All three of these enzymes can function across a broad pH range, from just below neutral to very alkaline. Put together, these traits make all these pectinases excellent candidates for use with different process requirements.

**FIG 6:**
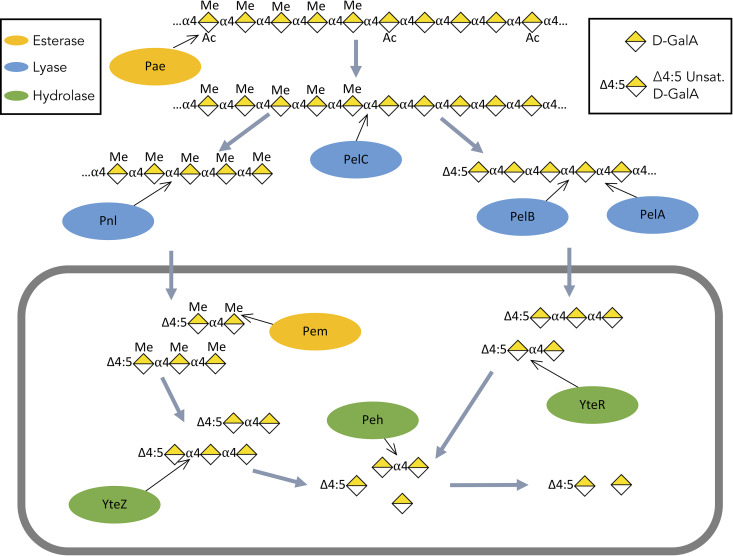
Emended model of homogalacturonan deconstruction in Paenibacillus amylolyticus 27C64. Black arrows indicate the sites targeted by each enzyme. Carbohydrates are represented using the standard nomenclature for glycans.

These findings also have broader implications for bacterial physiology. Most of the HG deconstruction enzymes known in Gram-positive bacteria have been studied in isolation and not in the context of a system (28). Our model of HG deconstruction in P. amylolyticus (Fig. 6) is therefore an important contribution to the overall understanding of diverse pectin deconstruction systems. Also, the unusually high activity on methylated pectin that was previously noted for PelA and PelB (24) has been specifically noted in another pectate lyase from Bacillus subtilis (29) and in a homologue of PelB from another species within the genus *Paenibacillus* (30). This suggests that the model presented here may be conserved in other Gram-positive bacteria and that this work may be able to serve as a resource to facilitate the understanding of related systems.

Future work should directly evaluate the utility of enzymes from this system, especially PelB, PelC, and Pnl, in industrial processes requiring neutral or alkaline conditions, such as fiber processing, coffee and tea fermentation, paper biobleaching, and cotton bioscouring. The conservation of this HG deconstruction system in other bacteria should also be explored, since similar systems may be important to environmental biomass degradation.

## MATERIALS AND METHODS

### Strains and culture conditions.

P. amylolyticus 27C64 was previously isolated from an insect hindgut (23) and was grown at 37°C with shaking in tryptic soy broth without dextrose (17 g tryptone, 3 g Soytone, 5 g NaCl, 2.5 g NaK_2_PO_4_ per liter) or on tryptic soy agar (15 g tryptone, 5 g Soytone, 5 g NaCl, 15 g agar per liter). Escherichia coli strains were grown at 37°C with shaking in LB (10 g tryptone, 5 g yeast extract, 10 g NaCl per liter) or on LB agar (including 15 g agar per liter) unless indicated otherwise. When antibiotics were necessary to maintain plasmid selection, ampicillin was used at a concentration of 100 μg/ml and chloramphenicol was used at 40 μg/ml (Sigma-Aldrich, St. Louis, MO). Terrific broth (24 g yeast extract, 12 g tryptone, 4 ml glycerol, 2.31 g KH_2_PO_4_, 12.54 g K_2_HPO_4_ per liter) was used to express some of the genes.

### Cloning.

The genes included in this study were analyzed for signal peptides by using SignalP 4.0 (31), Phobius (32), and PSORTb 3.0 (33) as described previously (25). The sequences of each enzyme included in this study are publicly available (25, 34). Each gene was amplified without its signal peptide (if present) in a 50-μl PCR mixture with high-fidelity Phusion DNA polymerase according to the manufacturer’s instructions (New England Biolabs, Ipswich, MA). Template genomic DNA was isolated from P. amylolyticus 27C64 with a Wizard Genomic DNA purification kit (Promega, Madison, WI), and oligonucleotide primers were provided by Integrated DNA Technologies (Coralville, IA). Primers included 20 bp of homology to a pET-21a-derived plasmid that included an N-terminal tag composed of an 8× His sequence, the Pyrococcus furiosus rubredoxin gene, a FLAG tag, and a tobacco etch virus (TEV) protease cleavage site. Genes were inserted into an AatII site immediately downstream of the TEV recognition sequence such that no non-native amino acids would remain after tag removal. Sequence- and ligation-independent cloning was used to assemble the plasmids as described previously, except that PCR products were gel purified in lieu of DpnI treatment and spin column purification (35). Plasmids were transformed into chemically competent E. coli DH5α prepared as described elsewhere (36), isolated with the ZR Classic plasmid purification kit (Zymo Research, Irvine, CA), and sequenced to confirm that the insert was correct (Eton Bioscience, Research Triangle, NC).

### Protein expression and purification.

Plasmids were transformed into different strains of E. coli for expression: BL21(DE3) was used for PelA, PelB, PelC, and Pnl, while Rosetta 2(DE3) (MilliporeSigma, Burlington, MA) was used for PelD. E. coli KRX (Promega, Madison, WI) was used for YteR, YteZ, and YesR. Each of the five lyases was grown in 100 ml of Terrific broth in 500-ml baffled Erlenmeyer flasks at 37°C with shaking. When cultures reached an optical density of 0.8 to 1.0, they were transferred to a shaker set at 19°C, allowed to equilibrate for 30 min, and then induced with 1 mM isopropyl-β-d-thiogalactopyranoside (IPTG; Gold Biotechnology, St. Louis, MO) and left overnight. The strains carrying the three cytoplasmic hydrolases were grown in 10 ml of ZYP-5052 autoinduction medium (37) supplemented with 0.1% rhamnose (Sigma-Aldrich, St. Louis, MO) plus 1 mM IPTG. Strains were allowed to grow at 25°C with shaking for 24 h. Cells from each culture were harvested by centrifugation and were stored at –20°C until needed.

The cell paste was resuspended in 5 ml of equilibration buffer (50 mM phosphate [pH 7.6], 300 mM NaCl) per g of cell paste and was sonicated on ice. The lysate was clarified by centrifugation at 17,000 rpm using a JA-20 rotor in a Beckman (Brea, CA) floor centrifuge and was then filtered through a 0.45-μm polyethersulfone (PES) membrane filter (MilliporeSigma, Burlington, MA) and bound to Ni-nitrilotriacetic acid (NTA) resin. The larger samples were purified using an ÄKTA start (GE Life Sciences, Chicago, IL) system with a 5-ml bed of Ni-NTA resin (Sigma-Aldrich, St. Louis, MO) in a GE XK 16/40 column. Smaller samples were purified using Ni-NTA spin columns (Qiagen, Hilden, Germany). All samples were washed with 10 or more column volumes of equilibration buffer with 10 mM imidazole and were eluted with elution buffer (50 mM phosphate [pH 7.6], 300 mM NaCl, 250 mM imidazole, 10% glycerol). Hydrolases were dialyzed into 25 mM Tris (pH 7.2) with 25 mM NaCl and were stored at 4°C (the tags were not removed). Lyases were dialyzed into 10 mM Tris (pH 7.5) with 10 mM NaCl. Tag cleavage reactions were set up by bringing the buffer strength up to 50 mM, adding 1 mM Tris(2-carboxyethyl)phosphine hydrochloride (TCEP), and adding the amount of TEV protease recommended for the P1′ amino acid of the construct (38). After tag cleavage overnight at 4°C, uncut protein and free tag were removed by passing the samples over Ni-NTA resin equilibrated with 50 mM phosphate (pH 8.0) with 300 mM NaCl. Then the samples were dialyzed into 25 mM Tris (pH 7.2) with 25 mM NaCl and 10% glycerol and were frozen at –20°C. The purity of all samples was evaluated using SDS-PAGE as described elsewhere (39).

### Enzyme assays.

Lyase activity was measured by monitoring the increase in absorbance at 232 nm as unsaturated products were formed (40). Diluted enzymes were stored on ice, and 5 μl was added to 995 μl of substrate solution just before data were collected. Reaction mixtures above or below room temperature were allowed to equilibrate at the target temperature for 5 min in a Peltier-thermostatted cuvette holder before the enzyme was added. A Cary 60 spectrophotometer (Agilent, Santa Clara, CA) was used to monitor the absorbance for 1 min and to determine the slope. Activity in international units (IU; expressed in micromoles per minute) was calculated using published molar extinction coefficients (40). Substrate solutions for determining the optimum reaction conditions of pectate lyases contained 0.2% polygalacturonic acid (PGA) and 0.5 mM CaCl_2_ in 100 mM buffer. Acetate buffers were used for pH values of <6, HEPES for pH 6 to 7, Tris for pH 7 to 9, and glycine-NaOH for pH values of >9. Optimum calcium concentrations and temperatures were determined at each enzyme’s optimal pH. Pectin lyase optimization used the same buffers but contained 0.05% esterified citrus pectin (Sigma-Aldrich, St. Louis, MO) and no calcium. For substrate specificity experiments, 0.5 mg of each substrate/ml was dissolved in 50 mM Tris (pH 8.0) with 1 mM CaCl_2_. Reaction mixtures for reaction progress curves contained only 0.25 mg of each substrate/ml.

GH105 hydrolase activity was monitored by the decrease in absorbance at 232 nm. As the terminal unsaturated carbohydrates are released, the ring-opening reaction reduces the absorbance (41). To generate unsaturated oligogalacturonides, 0.5 mg/ml PGA or esterified citrus pectin in 50 mM phosphate buffer (pH 6.9) was digested with PelB and PelC or with Pnl, respectively. Digestion reaction mixtures included enzymes at a 10 nM concentration, and digestion was allowed to proceed for 24 h at room temperature before products were heat inactivated by boiling for 5 min. Hydrolase assay reaction mixtures contained 2 μl of each enzyme in 398 μl of substrate solution, and reactions were monitored in 1-mm-path-length quartz cuvettes due to the high starting absorbance.

For all assays, specific activity was calculated by dividing the activity in international units per milliliter by the concentration of the enzyme solution added to the assay. Enzyme concentrations were determined by measuring the absorbance at 280 nm using a NanoDrop Lite spectrophotometer (Thermo Fisher, Waltham, MA) and the molar extinction coefficients predicted by ProtParam (42).

### Kinetics.

The activity of each enzyme was determined in triplicate at a range of substrate concentrations from 0.01 to 2.0 mg/ml. Reactions were performed at room temperature in 50 mM Tris (pH 8.0) with 1 mM CaCl_2_ and were monitored for 1 min. Enzyme concentrations in the reaction mixtures were 5 nM for PelB, 10 nM for PelD and Pnl, and 50 nM for PelA and PelC. Nonlinear least-squares minimization fitting to the Michaelis-Menten model in R was used to calculate the kinetic parameters.

### Analysis of pectins.

The degree of methylation and degree of acetylation of each pectin were determined by releasing the esterified groups with a NaOH-isopropanol mixture and quantifying the amount of acetate and methanol released using an Aminex HPX-87H high-performance liquid chromatography (HPLC) column (Bio-Rad, Hercules, CA) (43). Galacturonic acid content was determined by the *m*-hydroxydiphenyl colorimetric uronic acid assay with galacturonic acid as the external standard (44). This assay was scaled down linearly so that the volumes were appropriate for a PCR plate that could be heated in an aluminum heat block, and samples were transferred to a standard microtiter plate for reading of the absorbance. The molecular weight values (*M*_p_, *M*_n_, *M*_w_) and polydispersity index (PI) of each pectin were determined using a high-performance size exclusion chromatography method described elsewhere (45). Shodex P-82 narrow-polydispersity pullulan molecular weight standards (Showa Denko, Tokyo, Japan) were used to generate a calibration curve.

### Statistical analysis.

JMP Pro 14 was used to calculate multiple linear regressions that described the activity of each lyase as a function of substrate characteristics. Enzyme activity was normalized as a percentage of the maximum activity for each enzyme. To enable comparison of parameter estimates, values of independent variables were standardized by subtracting the mean of each variable from the individual values for that independent variable and dividing by the standard deviation. None of the enzymes examined were active on rhamnogalacturonan substrates, which were enzymatically treated to remove HG, so those compounds were excluded from the analysis.

### Analysis of reaction products.

Products from pectin digestion reactions were separated using a Shimadzu (Kyoto, Japan) Prominence HPLC system equipped with a TSKgel G3000PW_XL_ high-performance size exclusion chromatography column and a TSKgel PW_XL_ guard column (Tosoh, Tokyo, Japan). The mobile phase was 50 mM phosphate buffer, pH 6.9, with 100 mM NaCl, and the column oven was maintained at 40°C. Products were detected by the absorbance at 232 nm, and the UV detector cell was maintained at 40°C. A galacturonic acid standard from Sigma (St. Louis, MO) and di- and tetragalacturonic acid standards from Toronto Research Chemicals (Toronto, Canada) were used as markers.

### Data availability.

The genome sequence of P. amylolyticus 27C64 is available in GenBank under accession no. RIAS01000000. All gene and protein sequences for enzymes described in this study can be located by the names used in this report.

## Supplementary Material

Supplemental file 1
