# Supplementary material for: The Homogalacturonan Deconstruction System of Paenibacillus amylolyticus 27C64 Requires No Extracellular Pectin Methylesterase and Has Significant Industrial Potential
Source: Appl Environ Microbiol. 2020 Jun 2;86(12):e02275-19. doi: 10.1128/AEM.02275-19 (PMC7267202; doi:10.1128/AEM.02275-19)
Supplement: Supplemental file 1 [file AEM.02275-19-s0001.pdf]

Table S1 Inventory and characteristics of pectins used in this study.

Code	Substrate	Manu- facturer	Product No.	Lot No.	DM (%)	DA (%)	GalA (wt %)	Mp (kDa)	Mn (kDa)	Mw (kDa)	PI
Polygalacturonic acids											
PGA-A	Polygalacturonic acid, orange	Sigma	P3889	BCBB4735	0.0	1.2	89	172	89.9	144	1.61
PGA-B	Polygalacturonic acid, orange	Sigma	P3889	BCBD8707V	2.2	0.8	89	172	90.5	144	1.60
PGA-C	Polygalacturonic acid, orange	Sigma	P3889	BCBV4304	0.0	1.2	90	169	106	148	1.40
PGA-D	Polygalacturonic acid, orange	Sigma	P3889	BCBV4304	1.0	1.2	90	169	107	148	1.38
Rhamnogalacturonans											
RG_P-A	Rhamnogalacturonan I, potato	Megazyme	P-RHAM1	141102a	1.9	2.0	56	3.04	38.2	97.7	2.56
RG_S	Rhamnogalacturonan, soy	Megazyme	P-RHAGN	20202b	0.0	2.7	45	203	173	291	1.68
Unmodified pectins											
CP_ICN	Pectin, citrus	ICN	102587	4213A	34.4	2.6	65	0.35	70.8	153	2.16
CP_Sigma	Pectin, citrus peel	Sigma	P9135	SLBS8828	54.9	2.5	81	166	124	175	1.42
AP_Sigma	Pectin, apple	Sigma	93854	BCBS3576	58.1	3.4	80	170	108	161	1.49
CP_20_34-A	Pectin, 20-34% esterified, citrus	Sigma	P9311	077K1583	21.9	1.8	74	160	89.2	153	1.71
CP_20_34-B	Pectin, 20-34% esterified, citrus	Sigma	P9311	040M1178V	15.9	1.6	75	95.1	81.3	136	1.67
CP_55_70-A	Pectin, 55-70% esterified, citrus	Sigma	P9436	018K1649	44.2	2.1	85	170	121	202	1.67
CP_55_70-B	Pectin, 55-70% esterified, citrus	Sigma	P9436	051M1376V	53.8	2.5	76	170	129	203	1.58
CP_85_A	Pectin, 85% esterified, citrus	Sigma	P9561	110M1409V	77.1	1.5	83	160	90.3	136	1.51
CP_85_B	Pectin, 85% esterified, citrus	Sigma	P9561	BCBS3782V	81.7	1.4	87	164	96.7	143	1.48
CP_85_C	Pectin, 85% esterified, citrus	Sigma	P9561	BCBS3782	80.3	1.4	91	164	97.5	145	1.49
CP_90	Pectin, 90% esterified, citrus	Sigma	P9561	037K1272	33.8	1.2	98	170	74.8	128	1.71

DM – Degree of Methylation; *DA* – Degree of Acetylation, *GalA* – Galacturonic Acid, *Mp* – Peak Molecular Weight, *Mn* – Number-average Molecular Weight, *Mw* – Weight-average Molecular Weight, *PI* – Polydispersity Index.

Table S2 Results of linear models describing HG lyase activity as a function of pectin characteristics.

	**PelA**			**PelB**			**PelC**			**PelD**			**Pnl**		
	**Est.**	***t***	***p***	**Est.**	***t***	***p***	**Est.**	***t***	***p***	**Est.**	***t***	***p***	**Est.**	***t***	***p***
**DM**	-1.1	-0.66	0.5113	1.1	0.93	0.3566	16.6	8.4	<.0001*	5.8	3.06	0.0040*	33.9	58.25	<.0001*
**DM^2**	-20.1	-8.23	<.0001*	-34.4	-20.18	<.0001*	-16.9	-5.83	<.0001*	-28.1	-10.06	<.0001*	7.3	8.53	<.0001*
**DA**	7.6	2.86	0.0068*	1.2	0.64	0.5251	-1.1	-0.34	0.7353	13.1	4.33	0.0001*	0.6	0.67	0.5087
**Mn**	-27.3	-10.32	<.0001*	-17.3	-9.41	<.0001*	1.1	0.35	0.7293	-17.1	-5.66	<.0001*	-5.3	-5.79	<.0001*
**GalA**	29.6	9.74	<.0001*	14.1	6.66	<.0001*	1.9	0.53	0.6008	26.9	7.75	<.0001*	8.4	7.88	<.0001*
**R^2**	0.860			0.948			0.761			0.867			0.992		

All tests had 39 degrees of freedom. *Est* - Parameter estimate, *DM* – Degree of Methylation; *DA* – Degree of Acetylation, *GalA* – Galacturonic Acid, *Mn* – Number-average Molecular Weight

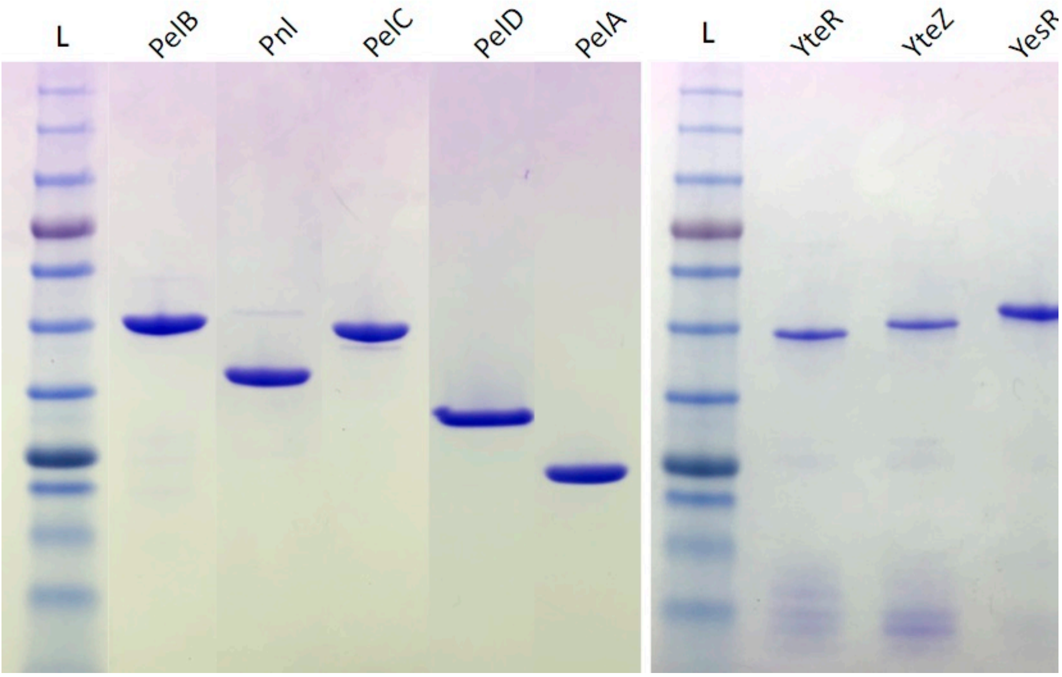

Figure S1. SDS-PAGE analysis of purified proteins included in this study. Intermediate purification steps have been cropped out of the leftmost six lanes but the position of each band relative to the ladder has been preserved. Gold Bio Bluestain2 was used as the molecular weight marker (indicated by lanes marked 'L').

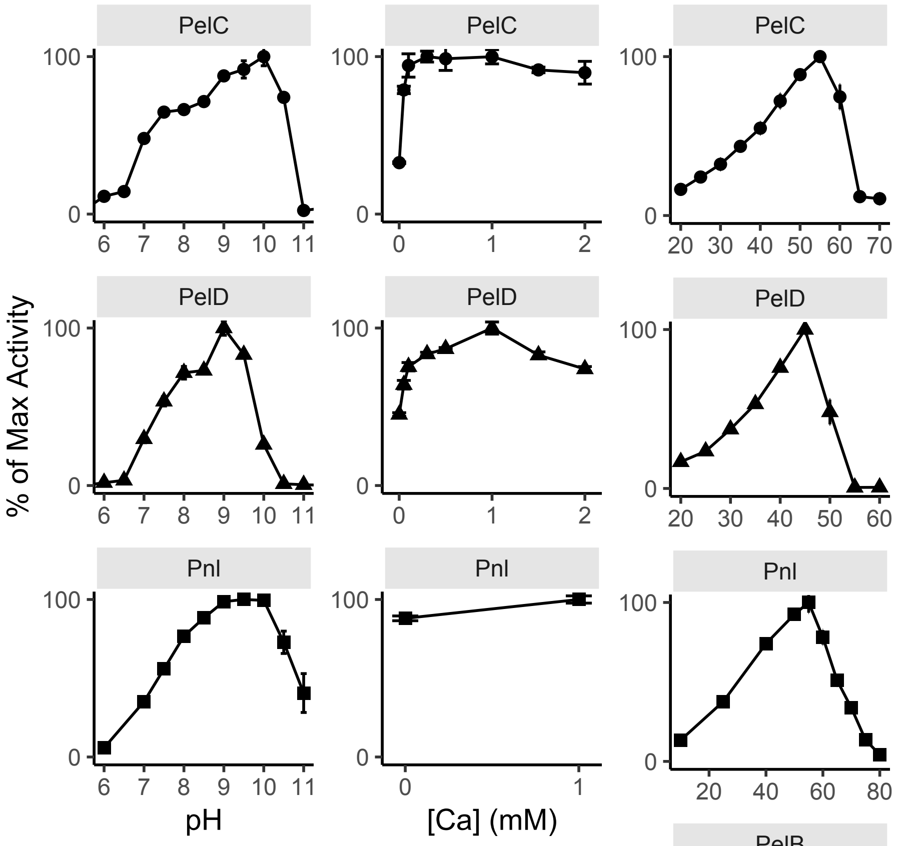

	PelC	PelD	Pnl	PelB
pH	10.0	9.0	9-10	--
[Ca] (mM)	0.1-1	1.0	N/A	--
Temp (°C)	55	45	55	70

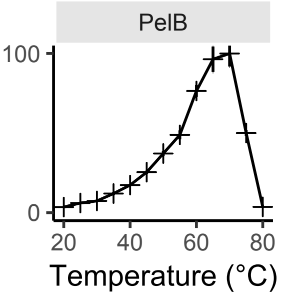

Figure S2. Optimum reaction conditions for three new pectic lyases and updated temperature profile for PelB. Each point and error bar represent the mean and standard deviation of three replicates. Individual optima are summarized in the table (inset). The optimal values were determined sequentially: pH first, calcium second, and temperature last. The temperature profile of PelB was re-evaluated because new equipment made accurate measurement at elevated temperatures possible.

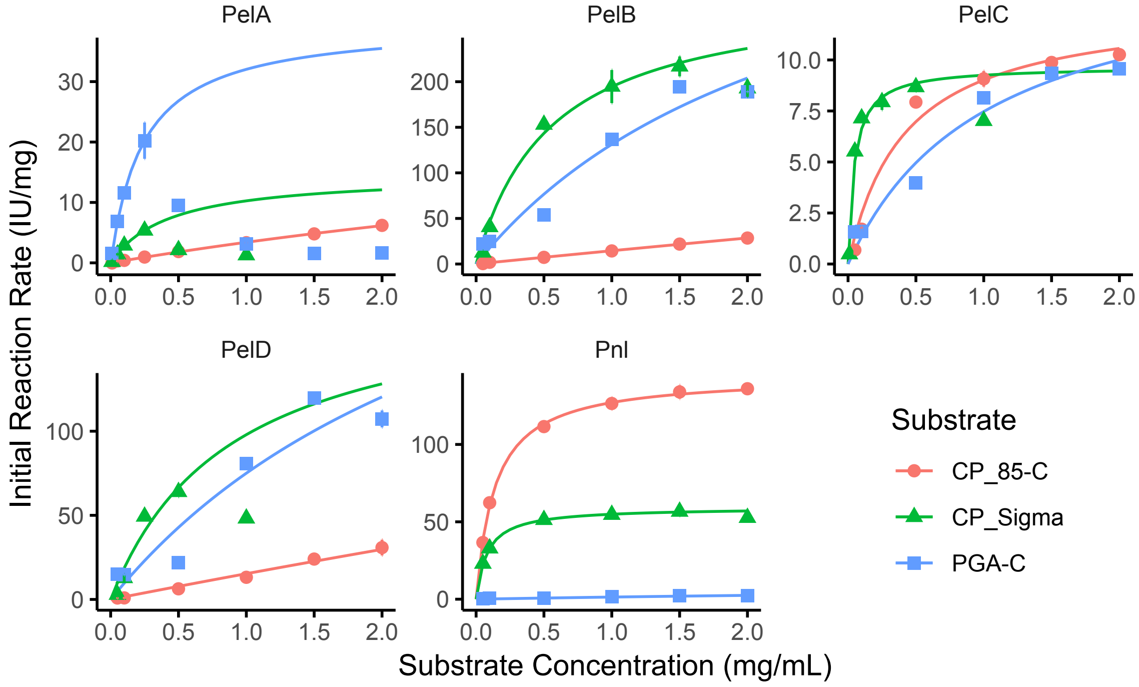

Figure S3. Activity of each extracellular lyase on different concentrations of three different pectins. Each point represents the initial reaction rate (n = 3). Kinetic parameters were determined by non-linear least-squares minimization fitting to the Michaelis-Menten equation. Lines represent theoretical activity based on calculated K_m and V_{max} values.

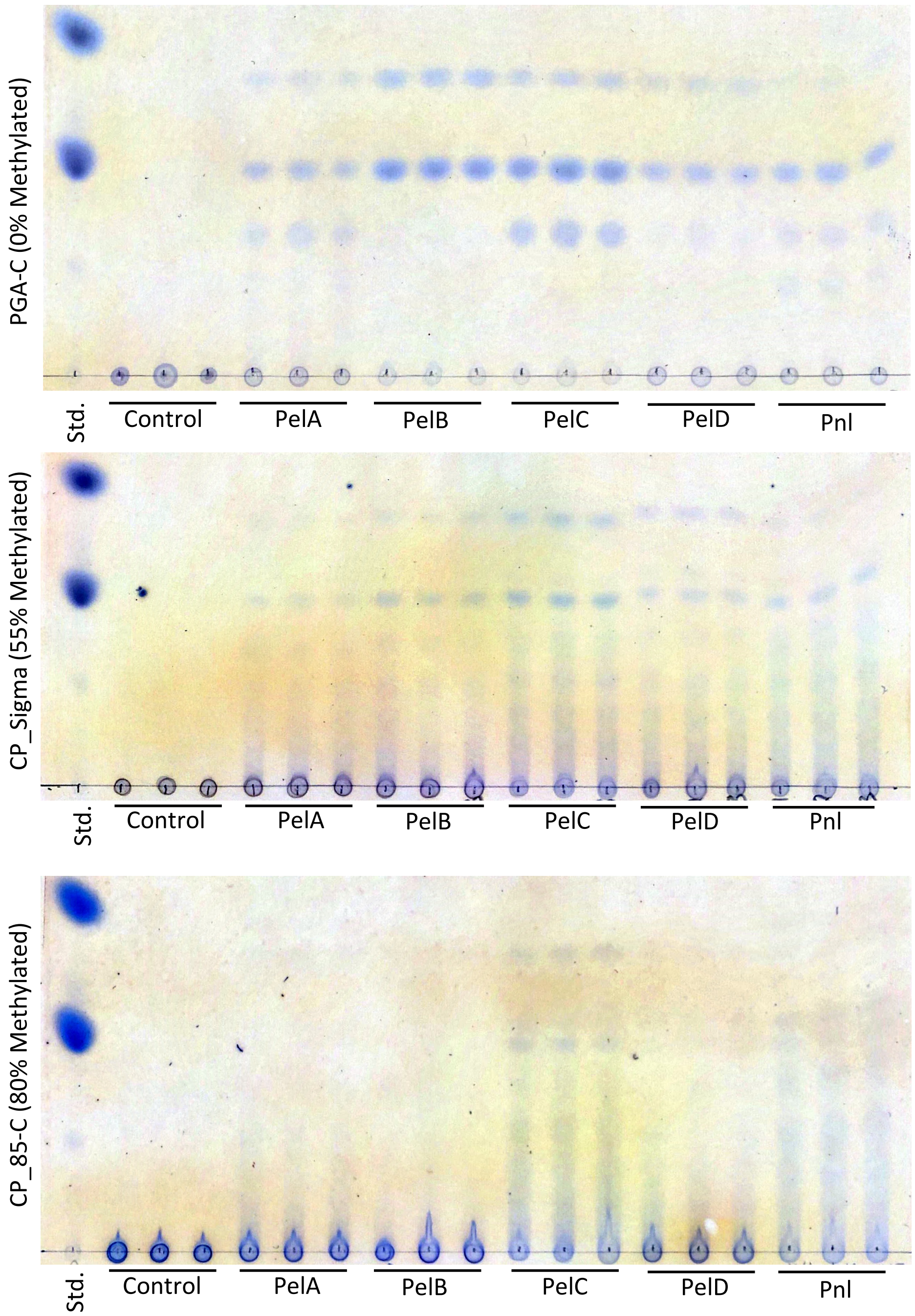

Figure S4. Thin-layer chromatography analysis of digestion products released from three different pectins by each extracellular lyase. All reactions were allowed to run to completion (24 hours). The leftmost sample on each plate is a mixed mono- and di-galacturonic acid standard. Labels below each sample identify replicate digestion reactions, and labels on the left of each plate indicate the substrate used for those reactions. TLC separations were performed as previously described (1).

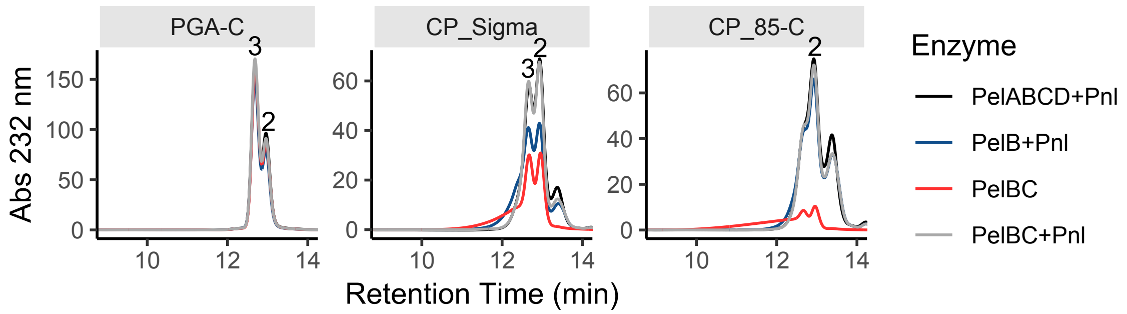

Figure S5. Products released by selected lyase combinations on three different pectins. Products were separated with high-performance size exclusion chromatography and elution profiles were monitored as the absorbance at 232 nm. Labels over peaks represent the degree of polymerization associated with that peak and were determined by comparison to oligogalacturonide size standards.

REFERENCES

1. Pedrolli DB, Carmona EC. 2014. Purification and characterization of a unique pectin lyase from Aspergillus giganteus able to release unsaturated monogalacturonate during pectin degradation. Enzyme Res 2014:353915.
